# The Arrowhead Ministernotomy with Rigid Sternal Plate Fixation: A Minimally Invasive Approach for Surgery of the Ascending Aorta and Aortic Root

**DOI:** 10.1155/2014/681371

**Published:** 2014-11-18

**Authors:** Mark J. Russo, John Gnezda, Aurelie Merlo, Elizabeth M. Johnson, Mohammad Hashmi, Jaishankar Raman

**Affiliations:** ^1^Barnabas Heart Hospitals, Newark Beth Israel Medical Center, 201 Lyons Avenue, Suite G5, Newark, NJ 07112, USA; ^2^Cardiovascular Clinical Research Unit, Barnabas Heart Hospitals, Newark, NJ 07112, USA; ^3^Section of Cardiac and Thoracic Surgery, University of Chicago, Chicago, IL 60637, USA; ^4^Division of Cardiac Surgery, Rush University, Chicago, IL 60612, USA

## Abstract

*Background*. Ministernotomy incisions have been increasingly used in a variety of settings. We describe a novel approach to ministernotomy using arrowhead incision and rigid sternal fixation with a standard sternal plating system. *Methods*. A small, midline, vertical incision is made from the midportion of the manubrium to a point just above the 4th intercostal mark. The sternum is opened in the shape of an inverted T using two oblique horizontal incisions from the midline to the sternal edges. At the time of chest closure, the three bony segments are aligned and approximated, and titanium plates (Sternalock, Jacksonville, Florida) are used to fix the body of the sternum back together. *Results*. This case series includes 11 patients who underwent arrowhead ministernotomy with rigid sternal plate fixation for aortic surgery. The procedures performed were axillary cannulation (*n* = 2), aortic root replacement (*n* = 3), valve sparing root replacement (*n* = 3), and replacement of the ascending aorta (*n* = 11) and/or hemiarch (*n* = 2). Thirty-day mortality was 0%; there were no conversions, strokes, or sternal wound infections. *Conclusions*. Arrowhead ministernotomy with rigid sternal plate fixation is an adequate minimally invasive approach for surgery of the ascending aorta and aortic root.

## 1. Introduction

Ministernotomy incisions have been increasingly used in a variety of cardiac surgery settings in an attempt to reduce surgical trauma and hasten patient recovery [1–5]. One of the first described ministernotomy techniques was the T incision [6]. However, despite offering superior exposure, that technique has been avoided after reports of poor healing of the transverse portion [7]. Currently, the J incision technique is the most widely used, although this technique limits exposure. Other less common techniques, such as the “upper V-type” ministernotomy in the second intercostal space, also involve limited exposure [8].

Here we describe a novel approach to ministernotomy by employing two simple but, in our experience, important modifications to the T incision technique. Firstly, the horizontal limb of the inverted T sternotomy is altered to an arrowhead shape. Secondly, rigid sternal fixation by a standard sternal plating system is used to maximize exposure, while limiting risk of sternal complications.

## 2. Methods

### 2.1. Preoperative Assessment

Preoperatively, a CT scan is obtained to assess relative location of the aorta to the sternum. If the aortic root is lower than the 4th intercostal space, we favor a full sternotomy.

### 2.2. Procedure

**Figure 1:**
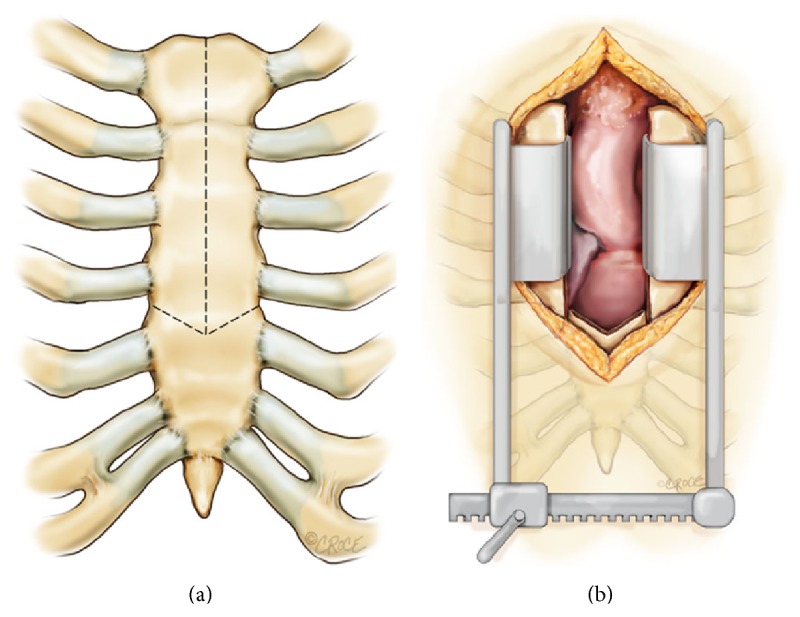
Markings made prior to opening the sternum (a); typical access through an arrowhead ministernotomy incision (b).

A small vertical skin incision is made from the midportion of the manubrium to a point just above the 4th intercostal mark. A standard sternal saw is used to open the sternum vertically, starting at the midline of the suprasternal notch and finishing at the horizontal limb of the inverted T (Figure 1). An oscillating saw with a narrow blade is then used to cut the limbs of the arrowhead from the midline to the sternal edge of the intercostal spaces. This configuration allows for a visualization of the aortic root, the right atrial appendage, and the main pulmonary artery (Figure 1). The exposure can be further enhanced with the use of right axillary artery cannulation instead of central cannulation. This modification allows for better access to the arch and better antegrade cerebral perfusion. Cardioplegia is administered using a coronary ostial balloon tipped cannula (Vitalcor, Westmont, Illinois). Next, a left ventricular vent is placed through the right superior pulmonary vein. The surgery on the aorta is then performed in the standard fashion.

### 2.3. Chest Closure

**Figure 2:**
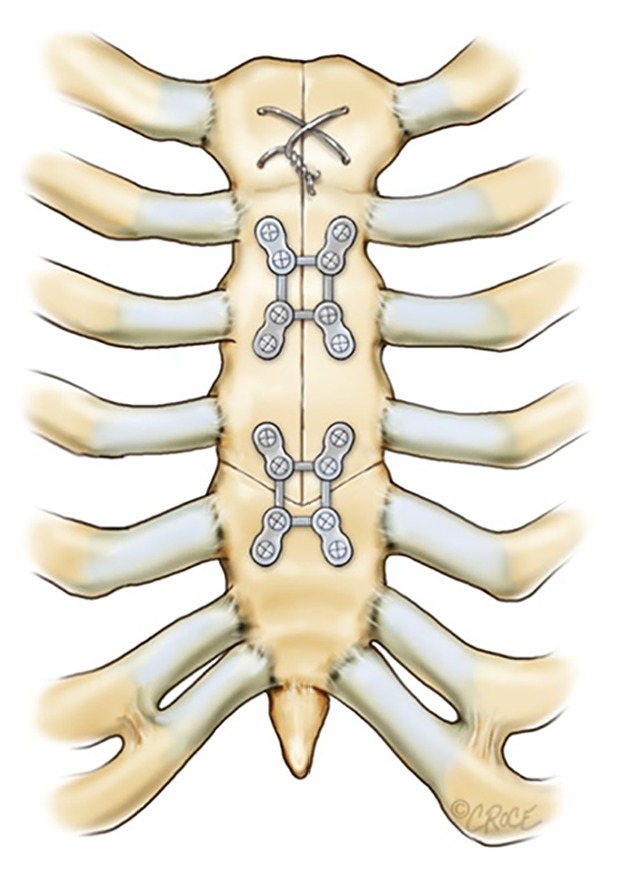
Arrowhead sternotomy closure with a rigid sternal fixation system.

At the time of chest closure, care is taken to align the sternal fragments, ensuring that there is no gap between the bony edges. Bone reducing forceps are used to carefully align the bony edges accurately. Firstly, a smaller pair of bone approximating forceps is placed in the 2nd intercostal space to get good vertical alignment of the body of the sternum. Secondly, obliquely placed larger forceps are placed spanning from the lateral sternal edge at the 3rd intercostal place to the opposite sternal edge at the 5th interspace in order to align the lower sternal fragment with the upper two sternal fragments. Finally, simple wires, or a figure of 8 wires in the manubrium, are used to bring the sternum together. Rostrocaudal alignment can be achieved by placing either parasternal wires or a stout suture that extends from one intercostal space above to one space below the transverse portion of the sternotomy bilaterally. Alignment is greatly enhanced, because the segments have a natural tendency to fit together due to the arrowhead configuration. Lateral displacement of the segments is inhibited by the oblique angle of the transverse cuts. Once all three bony segments are well aligned and approximated, titanium plates (Sternalock, Biomet Microfixation, Jacksonville, Florida) are used to fix the body. One “X” shaped plate is used across the arrowhead junction and a second is used to fix the body of the sternum (Figure 2). An additional “L” or “box” plate can be used as needed.

## 3. Results

This experience includes 11 patients who underwent an arrowhead approach to aortic surgery. Procedures included axillary cannulation (*n* = 2), aortic root replacement (*n* = 3), valve sparing root replacement (*n* = 3), and replacement of the ascending aorta (*n* = 11) and/or hemiarch (*n* = 2). Three patients needed a reoperative sternotomy. Tables 1 and 2 summarize preoperative risk factors and postoperative outcomes, respectively. In this early series, there were no conversions, strokes, or sternal wound infections. 30-day mortality was 0%. There was one reoperation for bleeding, which occurred on postoperative day 5, after heparin was started for atrial fibrillation.

## 4. Discussion

In this early series of patients undergoing aortic surgery, we have found the arrowhead modification to the T incision approach to be a safe alternative approach to ministernotomy. This approach has the benefit of combining the excellent surgical exposure of a full sternotomy with the excellent clinical outcomes (superior sternal stability and wound healing) of ministernotomies.

Crucial components of this approach include preoperative assessment by computed tomography to ensure that the anatomy is amenable to the incision, the arrowhead sternotomy itself, and the use of a rigid sternal fixation system. While other studies have demonstrated that, in the setting of a full sternotomy, the inferior portion of the sternum is at a greater risk for dehiscence [9], this approach preserves the lower portion of the sternum. Moreover, in our experience, the arrowhead incision allows for more reliable apposition and alignment of the sternal fragments. Our experience confirmed previous reports that the addition of a rigid closure system also offers greater sternal stability [10, 11].

## 5. Conclusion

Arrowhead ministernotomy with rigid sternal plate fixation is an adequate minimally invasive approach for surgery of the ascending aorta and aortic root. This approach has the benefit of combining the excellent surgical exposure of a full sternotomy, with the excellent outcomes of ministernotomies.

## Figures and Tables

**Table 1:** Preoperative risk factors.

Risk factor	*N*	%
Smoking history	1	9.1
Cerebral vascular accident	0	0
Diabetes mellitus	1	9.1
Previous cardiac surgery	3	27.3
Dialysis	0	0

**Table 2:** Postoperative outcomes.

Outcome measure	*N*	%
Mortality	0	0
Reoperation for bleeding	1	9.1
Stroke	0	0
Renal failure	0	0
Atrial fibrillation	2	22.2
Deep sternal wound infection	0	0
